# Contributions of major smoking-related diseases to reduction in life expectancy associated with smoking in Chinese adults: a cross-sectional study

**DOI:** 10.1186/1471-2458-13-1147

**Published:** 2013-12-09

**Authors:** Wei Han, Jingmei Jiang, Junyao Li, Xianjia Zeng, Xiaonong Zou, Yanping Wu, Yuanli Chen, Ping Zhao, Lei Hou, Haiyu Pang, Boqi Liu

**Affiliations:** 1 Department of Epidemiology and Biostatistics, Institute of Basic Medical Science, Chinese Academy of Medical Sciences & School of Basic Medicine, Peking Union Medical College Beijing, China; 2 The Cancer Institute/Hospital, Chinese Academy of Medical Sciences, 17 Pan Jia Yuan Nan Li, Beijing 100021, China

**Keywords:** Smoking, Smoking-related disease, Life expectancy, Chinese

## Abstract

**Background:**

Cigarette smoking is a prominent risk factor for a wide range of diseases. The current study aimed to evaluate the impact of smoking on deaths from major smoking-related diseases (neoplasms, vascular diseases and respiratory diseases) in Chinese adults by estimating the potential gains in life expectancy (LE) that would accrue from eliminating deaths from these diseases, and to determine the contribution of each disease to the reduction in LE associated with smoking.

**Methods:**

Two cohorts of Chinese smokers and non-smokers were constructed from a retrospective national mortality survey that had been conducted in 1989–1991 and included one million all-cause deaths among adults during 1986–1988 in 103 geographical regions. For each cohort, potential gains in LE by eliminating deaths from each major smoking-related disease were estimated. The contributions of each disease to smoking-associated reduction in LE were assessed using the LE decomposition approach.

**Results:**

Among the major smoking-related diseases, it was estimated that elimination of vascular diseases would provide the greatest potential gain in LE (years), regardless of smoking status. The gains for smokers versus non-smokers in populations of urban men, urban women, rural men and rural women aged 35 years were 3.5 vs. 4.3, 3.8 vs. 4.1, 2.4 vs. 3.0, and 2.6 vs. 2.9 years, respectively. Respiratory diseases contributed most to smoking-associated LE reductions in urban women, rural men and rural women of 43.6%, 46.4%, and 62.9%, respectively. In urban men, neoplasms contributed most to smoking-associated LE reduction, their contribution being estimated as 45.8%.

**Conclusions:**

Respiratory disease has the greatest influence on the LE reduction associated with smoking. Thus, smoking prevention could significantly reduce deaths from respiratory disease and improve LE.

## Background

Worldwide, many studies have shown that cigarette smoking increases the risk of death and is a prominent risk factor for a wide range of diseases such as neoplasms, respiratory diseases and vascular diseases [1-3]. Although these findings are meaningful from an etiological perspective, they fail to provide information regarding absolute public health effects. Life expectancy (LE) by smoking status, an effective measure of the health burden associated with smoking, has the advantage of being easy for both the general public and policymakers to interpret and understand.

The greatest challenge to estimating the impact of smoking-related diseases by determining LE in a large-scale representative cross-sectional study is the construction of cohorts of smokers and non-smokers, because of the difficulty in obtaining accurate information about smoking habits. Until now, only a few reports have directly estimated the reduction in LE caused by smoking [4-10]; most of these were based on cohort studies. Construction of cohort studies by smoking status to estimate mortality or disease incidence is time-consuming. Moreover, although such studies have found differences in LE according to smoking status, they have not identified which diseases make the greatest contributions to these differences. More data on the impact of different diseases and smoking status on mortality would provide useful data that could help public health providers to target efforts to attenuate the reduction in LE caused by smoking.

China, the world’s largest cigarette producer and consumer, has a particularly large health burden associated with smoking [11,12]. Therefore, there is an urgent need to provide an accurate assessment of the health burden caused by smoking and thus facilitate shaping of the most effective public health policies for smoking prevention and cessation.

During 1989–1991, a large nationwide retrospective study of smoking and mortality was conducted in a population of 27 million adults (35 years old or above) in 103 regions (24 major cities and 79 counties) of China [13]. The 24 cities were chosen non-randomly to cover a wide geographical region. Stratified random sampling techniques were used to choose 79 counties from 2000 counties whose cancer rates were recorded in the Chinese cancer atlas (yielding a population of 67 million). After this survey, a series of studies were conducted and their findings published, most estimating the impact of smoking on risk of death from a particular disease from an etiological perspective [13-15].

The present study aimed to further investigate the influences of various major smoking-related diseases on the reduction in LE, as well as the contribution of each disease to the smoking-related reduction in LE.

## Methods

The total population (approximately 27 million aged 35 years or older) from which the nationwide mortality survey was conducted during 1989–1991 was defined as the base population. Approximately 90% of subjects (almost one million) who had died in 1986–1988 were identified from the base population, as were their surviving spouses. Thus, the present study involved three populations: the base population, deceased subjects and their surviving spouses.

Causes of death were identified by reviewing the death certificates from the local Population Administrative Offices. If necessary, this information was supplemented by reviewing the medical records or by discussion (a few years after the death) with local health workers, family members or both. Underlying causes of death from the Population Administrative Offices records were coded according to the International Classification of Diseases, 9th Revision (ICD-9). Details of the study design, field survey methods and participants have been described elsewhere [13]. In the present study, major smoking-related causes of deaths included: neoplasms (ICD-9: 140–208), respiratory diseases (chronic obstructive pulmonary disease: 490–2, 496, 416–7; respiratory tuberculosis: 011, 012, 018; other respiratory diseases: rest of 460–519), and vascular diseases (stroke: 430–9; ischemic heart disease: 410–4; other vascular diseases: 390–409, 415, 418–429, 440–459); the remaining deaths were categorized as related to other causes.

The smoking habits of the deceased subjects were ascertained by interviewing their living spouses or other relatives to determine whether the subjects had smoked before 1980, a period of time before the onset of the disease that would have eventually caused death during 1986–1988. In addition, the smoking habits of the spouses of the deceased subjects were also determined during these interviews. Almost 100% of all identified households agreed to participate in the interview. In this investigation, non-smokers were defined as persons who had never smoked during their lifetime or who had only smoked infrequently at a young age.

To ascertain the smoking prevalence of the base population, a new design was employed in the present study, which used the prevalence of smoking of living spouses as a substitute for the smoking prevalence of the base population. The assumption of this design is that living spouses constituted an approximately random sample of the base population with similar smoking habits to those of the study base.

### Life tables and cause-deleted life table construction

Numbers of smokers and non-smokers in the base population stratified by age group were estimated by applying age-specific factors based on the proportions of smokers among spouses. During this process, 6% of the sample for whom no smoking information was available were excluded and the base population estimate was reduced by 10% because information on only 90% of deaths was available. The number of deaths per annum was calculated by dividing the total number of deceased subjects by three (number of study years). After these adjustments, the abridged life table technique was used to construct cause-deleted life tables for smokers and non-smokers separately by sex and area of residence (urban or rural), to assess the potential gains in LE by eliminating deaths from major smoking-related diseases [16].

### Cause-specific decomposition of LE reduction associated with smoking

As proposed by Preston et al. [17], the following equation (1) can be used to determine the difference in LE between smokers and non-smokers that is attributable to mutually independent death causes.

(1) $$e^{*}\left(35\right)-e\left(35\right)=\sum_{i=1}^{n}\sum_{x=35}^{w}(nL_{x,i}^{*}-nL_{x,i})\left(\frac{nL_{x,-i}^{*}+nL_{x,-i}}{2n}\right)$$

The following equation (2) can be used to decompose into two terms the difference in gained LE between smokers and non-smokers when deaths from a major smoking-associated disease have been eliminated.

(2) $$\begin{array}{l} D_{i}^{*}\left(35\right)-D_{i}(35)=\sum_{x=35}^{w}\left(nL_{x,-i}^{*}-nL_{x,-i}\right)(1-\frac{nL_{x,i}+nL_{x,i}^{*}}{2n})\\ \;-\sum_{x=35}^{w}\left(nL_{x,i}^{*}-nL_{x,i}\right)\left(\frac{nL_{x,-i}+nL_{x,-i}^{*}}{2n}\right) \end{array}$$

where, *e*(35) and e*(35) represent the LE for 35-year-old non-smokers and smokers, respectively; *D*_*i*_(35) and $D_{i}^{*}\left(35\right)$ represent the gain in LE at age 35 years for non-smokers and smokers, respectively, by eliminating cause of death *i*; and where *nL*_*x*,*i*_, $nL_{x,i}^{*}$, *nL*_*x*,- *i*_, and $nL_{x,-i}^{*}$ represent the person-years alive between ages x and x + 5 for non-smokers and smokers in life tables for causes *i* and –*i*, respectively.

The first term represents the change in survival from cause –*i* weighted by the cumulative probability of surviving from cause *i*; the second term represents the change in survival from cause *i* weighted by the cumulative probability of surviving from cause –*i:* the LE disparity between smokers and non-smokers is thus attributable to cause *i*[17]. Therefore, a change in significance of a certain cause of death results from the combined effects of a change in cause *i* and changes in causes other than *i*.

## Results

Of the base population, there were 1 059 804 deceased subjects (69.5% urban, 30.5% rural) and 307 853 living spouses (76.1% urban, 23.9% rural) aged 35 years or over in this study. The smoking prevalence among living spouses of the deceased subjects was 57.3% for men and 17.7% women in urban areas, and 64.1% and 8.6%, respectively, in rural areas. Table  1 presents the all-cause mortality rates by smoking status in the base population. The mortality rates among smokers were higher than among non-smokers for the age groups older than 50 years, regardless of sex and area of residence.

**Table 1:** Sex- and area-specific numbers of subjects in the base population and annual all-cause mortality rates by smoking status during 1986–1988

**Age group**	**Urban men**		**Rural men**		**Urban women**		**Rural women**	
**Smoker**	**Base population**	**Annual mortality rates**	**Base population**	**Annual mortality rates**	**Base population**	**Annual mortality rates**	**Base population**	**Annual mortality rates**
	**n**	**%**	**n**	**%**	**n**	**%**	**n**	**%**
35-39	1175320	0.1	431648	0.2	49392	0.1	20002	0.2
40-44	803444	0.2	355561	0.4	64568	0.2	19444	0.4
45-49	645155	0.4	298267	0.6	107316	0.4	30337	0.5
50-54	640748	0.9	277814	1.0	168475	0.7	34168	0.9
55-59	624586	1.4	245723	1.7	180234	1.1	31875	1.3
60-64	484225	2.4	193866	3.0	146389	1.9	29220	2.2
65-69	332614	4.2	138917	4.7	110927	3.3	22650	3.8
70-74	195844	7.6	89244	7.8	75321	5.9	17501	6.2
75-79	102896	11.5	48351	11.1	46026	9.0	10754	9.0
80+	51585	20.3	24237	21.0	32615	17.2	7876	17.3
**Non-smoker**								
35-39	681426	0.2	231406	0.3	1636345	0.1	589816	0.2
40-44	554413	0.2	187198	0.4	1226796	0.2	472812	0.3
45-49	455417	0.4	153790	0.6	920613	0.3	388111	0.4
50-54	454735	0.7	135293	0.9	898500	0.6	342135	0.7
55-59	396313	1.1	110449	1.5	764389	0.9	308307	1.0
60-64	329189	1.6	97838	2.3	583003	1.4	261815	1.6
65-69	229804	2.9	77465	3.3	418809	2.4	208706	2.5
70-74	155509	5.2	57105	5.2	301473	4.1	151593	4.4
75-79	94981	8.3	37820	6.8	192822	6.9	103161	6.1
80+	69195	15.0	24637	14.0	174729	14.4	77735	13.7

### Number of deaths from major smoking-associated diseases

**Figure 1:**
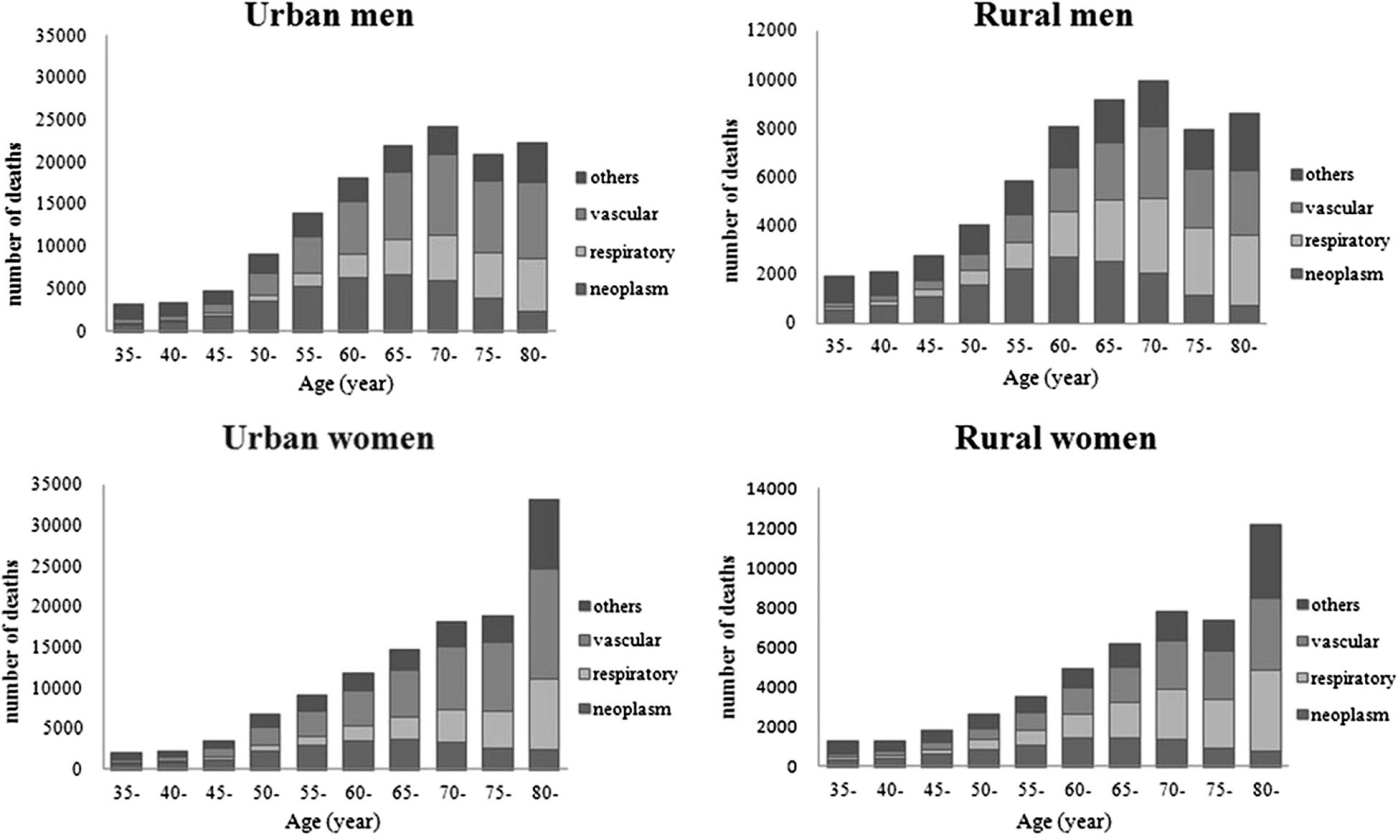
Distribution of deaths from smoking-associated diseases among various age groups by sex and area of residence.

Figure  1 presents the sex- and area-specific number of deaths from major smoking-related diseases for each age interval. Neoplasms, vascular diseases and respiratory diseases together account for the majority of deaths in Chinese adults. Almost 81.1% and 78.9% of the total deaths were attributable to one of these three diseases in urban men and women, respectively; in the rural population the corresponding proportions were lower at 75.3% and 75.1%, respectively.

Deaths from these smoking-related diseases were concentrated in different age groups. Deaths from neoplasms mostly occurred between 35 and 69 years and accounted for approximately two-thirds of all neoplasm-related deaths, regardless of sex and location. Compared with neoplasms, a larger proportion of deaths from vascular diseases or respiratory diseases occurred in later life, approximately two-thirds of deaths from respiratory or vascular diseases occurring at 70 years or older.

### Differences in LE according to smoking status and potential gains in LE related to elimination of major smoking-associated diseases

Tables  2 and 3 presents the estimated LE of smokers and non-smokers of different ages, as well as the potential gains in LE by the hypothesized elimination of deaths from each of the three diseases in subjects over 35 years old. In general, non-smokers had longer LEs than smokers, regardless of sex or area of residence. The all-cause LE (years) difference between 35-year-old smokers and non-smokers was 2.6 and 2.4, for urban men and women, respectively, and 2.3 and 2.9 for rural men and women, respectively.

**Table 2:** Life expectancy for smokers and non-smokers and potential gains in life expectancy (years) among men by eliminating deaths from a specific smoking-associated disease by smoking status

**Age group**	**Overall life expectancy**		**Gains in LE by eliminating neoplasms**		**Gains in LE by eliminating vascular diseases**		**Gains in LE by eliminating respiratory diseases**	
**Urban men**	**Smokers**	**Non-smokers**	**Smokers**	**Non-smokers**	**Smokers**	**Non-smokers**	**Smokers**	**Non-smokers**
35-39	36.59	39.20	3.15	2.41	3.48	4.29	1.99	1.49
40-44	31.84	34.53	3.09	2.34	3.47	4.27	2.00	1.48
45-49	27.17	29.90	3.00	2.25	3.45	4.25	2.00	1.47
50-54	22.70	25.43	2.82	2.09	3.39	4.19	2.00	1.46
55-59	18.60	21.22	2.53	1.86	3.26	4.06	2.00	1.45
60-64	14.78	17.24	2.14	1.59	3.08	3.84	1.97	1.41
65-69	11.38	13.49	1.69	1.30	2.81	3.54	1.91	1.35
70-74	8.47	10.23	1.21	0.95	2.45	3.08	1.78	1.25
75-79	6.26	7.55	0.74	0.61	1.95	2.40	1.54	1.07
80+	4.29	5.19	0.35	0.29	1.34	1.59	1.13	0.79
**Rural men**								
35-39	35.23	37.50	3.10	2.78	2.43	2.99	2.86	2.37
40-44	30.64	33.10	3.01	2.67	2.41	2.98	2.87	2.35
45-49	26.16	28.73	2.86	2.51	2.40	2.96	2.88	2.33
50-54	21.88	24.54	2.62	2.28	2.36	2.93	2.88	2.30
55-59	17.88	20.53	2.28	2.01	2.30	2.86	2.85	2.24
60-64	14.23	16.92	1.86	1.63	2.20	2.70	2.78	2.12
65-69	11.13	13.66	1.35	1.21	2.04	2.46	2.62	1.94
70-74	8.42	10.68	0.89	0.83	1.80	2.14	2.36	1.69
75-79	6.28	8.11	0.50	0.47	1.43	1.62	1.94	1.34
80+	4.18	5.39	0.25	0.22	1.01	1.13	1.39	0.95

**Table 3:** Life expectancy for smokers and non-smokers and potential gains in life expectancy (years) among women by eliminating deaths from a specific smoking-associated disease by smoking status

**Urban women**	**Overall life expectancy**		**Gains in LE by eliminating neoplasms**		**Gains in LE by eliminating vascular diseases**		**Gains in LE by eliminating respiratory diseases**	
	**Smokers**	**Non-smokers**	**Smokers**	**Non-smokers**	**Smokers**	**Non-smokers**	**Smokers**	**Non-smokers**
35-39	38.51	40.87	2.54	1.92	3.76	4.12	2.37	1.57
40-44	33.71	36.09	2.51	1.86	3.75	4.10	2.35	1.57
45-49	29.05	31.35	2.39	1.78	3.69	4.06	2.34	1.56
50-54	24.59	26.81	2.23	1.65	3.55	3.96	2.33	1.54
55-59	20.39	22.5	1.99	1.45	3.38	3.79	2.30	1.52
60-64	16.36	18.37	1.70	1.22	3.17	3.57	2.23	1.49
65-69	12.78	14.52	1.33	0.96	2.89	3.26	2.11	1.42
70-74	9.64	11.05	0.92	0.67	2.53	2.83	1.92	1.30
75-79	7.10	8.00	0.55	0.41	2.01	2.23	1.59	1.10
80+	4.78	5.29	0.25	0.20	1.38	1.47	1.17	0.82
**Rural women**								
35-39	37.18	40.11	2.32	2.07	2.60	2.89	4.09	2.59
40-44	32.61	35.48	2.24	2.00	2.55	2.86	4.03	2.57
45-49	28.14	30.90	2.08	1.90	2.50	2.82	4.00	2.54
50-54	23.80	26.48	1.88	1.74	2.42	2.75	3.95	2.49
55-59	19.76	22.28	1.64	1.51	2.31	2.63	3.81	2.43
60-64	15.91	18.27	1.34	1.26	2.16	2.48	3.66	2.32
65-69	12.45	14.59	1.02	0.94	1.98	2.25	3.42	2.16
70-74	9.51	11.22	0.67	0.64	1.71	1.94	3.04	1.90
75-79	7.08	8.35	0.41	0.37	1.34	1.48	2.39	1.49
80+	4.75	5.45	0.19	0.19	0.94	1.01	1.67	1.06

Overall, after the hypothesized elimination of deaths from respiratory diseases or neoplasms for smokers and non-smokers aged 35 years and above, the potential gains in LE (years) for smokers was greater than for non-smokers at every age, regardless of sex or area of residence. However, it did not follow the same pattern for vascular disease: non-smokers would potentially gain more in LE than smokers after elimination of deaths from vascular diseases.

### Contributions of major smoking-related diseases to the LE reduction associated with smoking

Column 3 of Table  4 presents the differences between smokers and non-smokers in the potential gains in LE according to each of three major smoking-related diseases and columns 4 and 5 show the results of decomposition of these differences, which correspond to the first term and second terms in equation (2). The values in column 3 are all positive except for that for vascular diseases: this indicates that the potential gains in LE related to vascular diseases are greater for non-smokers than for smokers. However, for all major smoking-related diseases the second terms in equation (2) are positive, indicating that the LE of smokers would have increased more than that of non-smokers if deaths from one of these three causes had been removed after taking changes in mortality from other diseases into account.

**Table 4:** Sex- and area-specific decomposition results of the difference in gained life expectancy (years) estimated by elimination of major smoking-associated diseases

**Cause of death**	**Gains in LE by eliminating a cause of death for non-smokers (**1**)**	**Gains in LE by eliminating a cause of death for smokers (**2**)**	**Difference (3)=(2)-(1) or (4) + (5)**	**First term in equation **2 (4)*	**Second term in equation **2 **(5)**^**Ŧ**^
**Urban men**					
Neoplasm	2.41	3.15	0.74	-0.45	1.19
Vascular disease	4.29	3.48	-0.81	-1.19	0.38
Respiratory disease	1.49	1.99	0.51	-0.40	0.91
**Rural men**					
Neoplasm	2.78	3.10	0.33	-0.51	0.84
Vascular disease	2.99	2.43	-0.56	-0.86	0.31
Respiratory disease	2.37	2.86	0.49	-0.55	1.04
**Urban women**					
Neoplasm	1.92	2.54	0.63	-0.28	0.90
Vascular disease	4.12	3.76	-0.36	-0.82	0.47
Respiratory disease	1.57	2.37	0.80	-0.26	1.06
**Rural women**					
Neoplasm	2.07	2.32	0.25	-0.39	0.64
Vascular disease	2.89	2.60	-0.29	-0.71	0.42
Respiratory disease	2.59	4.09	1.50	-0.33	1.83

*Changed significance of a smoking-associated cause of death that is estimated by changes in other causes of death in smokers and non-smokers.

^Ŧ^Life expectancy disparity between smokers and non-smokers that was attributable to a particular smoking-associated cause of death.

**Figure 2:**
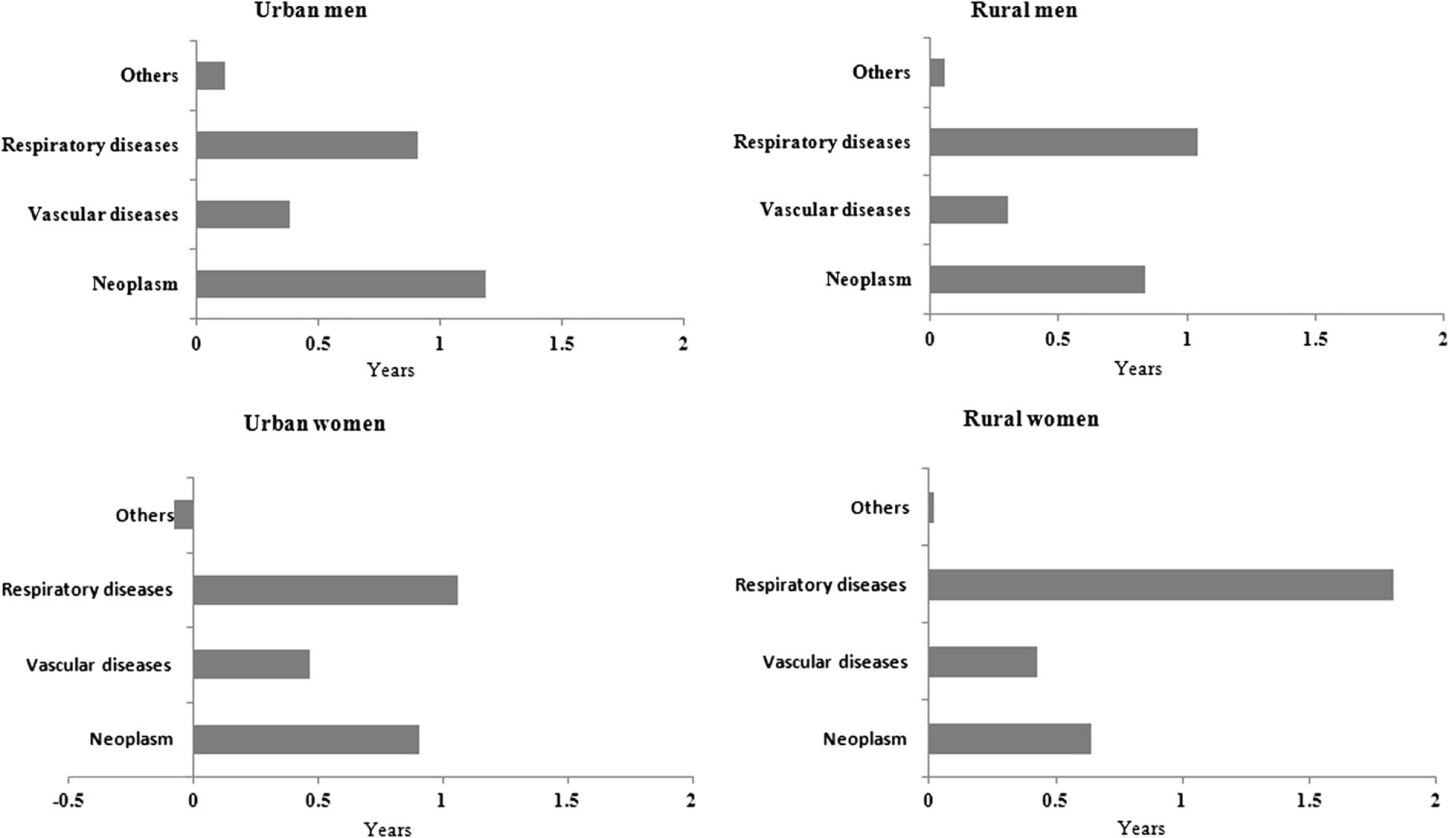
Sex- and area-specific decomposition results of life expectancy reductions associated with smoking.

Figure  2 shows the reduction in LE associated with smoking that was attributable to a particular smoking-associated cause of death (second term in equation [2]): the relative importance of a particular smoking-associated disease varied according to sex and area of residence. For urban men, neoplasms contributed most to the smoking-associated LE reduction, followed by respiratory diseases and vascular diseases (neoplasms: 45.8%, respiratory diseases: 35.0%, vascular diseases: 14.8%). For urban women, and rural men and women, the relative importance of the contributions of major smoking-related diseases followed a different pattern; the contributions of respiratory diseases, neoplasms and vascular diseases being 43.6%, 37.2% and 19.2%, respectively, in urban women; 46.4%, 37.4% and 13.6%, respectively, in rural men; and 62.9%, 22.0% and 14.5%, respectively, in rural women.

## Discussion and conclusion

Overall, we found that non-smokers lived more than 2 years longer than smokers. Respiratory disease, neoplasms and vascular disease all had a considerable influence in reducing LE associated with smoking, but to varying degrees. Nearly 78.6% of all deaths were attributable to one of the three major smoking-related diseases in Chinese adults; however, decomposition of the findings revealed that, together, the three major smoking-associated diseases contributed to 98.0% of the difference in LE between smokers and non-smokers.

Several aspects have to be considered when discussing the validity of these findings. First, the key new point in our study design is that the smoking prevalence of surviving spouses of deceased subjects was used as a substitute for the smoking prevalence of the base population; this allowed us to calculate the cause-specific LEs by smoking status. The basic assumption of this aspect of our study design is that the distribution of all causes of death in the base population is approximately random, as is the distribution of living spouses, who can therefore be regarded as a representative sample of the base population. We have confirmed the validity of this technique in several previous studies [14,15]. In addition, to support this assumption, we compared the smoking prevalence in living spouses with estimates of smoking prevalence from another national study and found high consistency between them [18]. Second, our figures for LEs regardless of smoking status are consistent with LEs reported for the same period in China [19], which also supports the validity of the smoking-specific LEs calculated on the basis of overall LE. Third, because we obtained the information about smoking habits from a retrospective mortality survey, this information may have been influenced by recall bias. Although findings from other studies have validated the fact that smoking-associated LE decreases with increasing numbers of smokers [8-10], we decided to minimize the influence of recall bias by simply dividing smoking information into two definite categories (smoker and non-smoker), since it is unlikely that many non-smokers would have been misreported as having been smokers. In addition, in a validity study in Shanghai in the early 1980s in which the spouses were the informants and both husband and wife reported their smoking habits, information obtained from the wife on the husband’s smoking habits was highly consistent with information provided directly by the husband [20].

Smoking harms almost every organ in the body: previous studies have demonstrated that many serious and fatal diseases are caused by smoking [3]. However, in our study, we mainly focused on neoplasms, respiratory diseases and vascular diseases, which together account for most deaths associated with smoking in China [21]. In addition, as a summary measure for health situation, LE is not sensitive when disease categories are defined in too much detail, especially when the diseases in question are associated with few deaths. Thus, the way we categorized smoking-related diseases in our study ensured sufficient numbers of deaths in each disease category.

We compared our results with those of studies conducted in other countries and found that the LE reductions associated with smoking in our study were similar to the findings of studies conducted in Japan [9,10]. However, in the United States, the difference in LE between 40-year-old smokers and non-smokers is reportedly approximately 6.9 years in men and 6.8 years in women [5]. Research conducted in Austria found the LE difference for 15-year-old men was 5.6 years [7]. A study conducted in the Netherlands showed that the LE difference for 40-year-old subjects was 7.5 years [8]. The magnitude of the reduction in LE associated with smoking in our study was relatively small compared with that reported by the above-mentioned studies. This disparity may be attributable to differences between different countries in the status of the smoking epidemic. In addition, the mortality for the whole population in China was relatively high [13]. Finally, the non-smoking group might have included some passive smokers and ex-smokers, which could have diluted the observed difference.

One interesting phenomenon in our findings that is not consistent with widely held knowledge is that, even though the death rate from vascular disease, which is a major category of smoking-related disease, was greater among smokers than non-smokers, we estimated that vascular diseases were associated with loss of more life years among non-smokers than among smokers. To explore this unusual phenomenon, we employed the LE decomposing techniques developed by Preston et al. These techniques show that the difference in years of life lost to a particular disease is equal to the amount of change attributable to that disease in a cause-decomposition, plus a simple additional term that reflects movements in other causes of death. In our study, decomposition showed that the contribution of vascular diseases to the difference in LE between smokers and non-smokers was positive (column 5 in Table  4), as was also true for neoplasms and respiratory diseases. However, other causes of death, which of course included neoplasms and respiratory diseases (column 4 in Table 4), made a greater contribution to LE. That is, although the mortality rate decreased more in smokers than in non-smokers after vascular diseases had been eliminated, the size of the reduction in mortality for respiratory diseases or neoplasms outweighed that for vascular diseases**.** In other words, the difference between smokers and non-smokers in life-shortening effects resulting from elimination of vascular diseases depends on the difference in mortality from respiratory diseases and neoplasms, which was greater than that from vascular diseases in our study.

The strengths of our study are as follows. First, rather than calculating LE for all-cause deaths, the framework of our new study design allowed assessment of the influence of a specific major smoking-related disease on LE reduction according to smoking status. This approach thus provides policymakers and the general public with intuitive and appropriate information concerning the adverse effect of smoking on health. Second, although smoking, an important risk factor for death from cardiovascular diseases, had indeed been proven to be associated with cardiovascular disease in China, the risk is quite low compared with that of neoplasms and respiratory disease according to a previous study based on this survey [13]. The present study further evaluated the contribution of cardiovascular disease and found that, after taking the change in mortality rates related to other causes of death into account, cardiovascular disease played a considerable role in the disparity in LEs.

Our study has some limitations. First and most importantly, it is based on a survey conducted two decades ago, since which time the general smoking prevalence has declined [22], which has probably influenced LE estimation by smoking status. However, because of the time lag between smoking and the onset of smoking-related disease, such decline in smoking prevalence would have had a limited influence on life expectancy changes during this period. Furthermore, we mainly estimated life years lost due to smoking and the contributions of major smoking related diseases (neoplasms, respiratory diseases and cardiovascular diseases) to the life years lost by the risk of deaths from those diseases; these have remained almost unchanged for the last two decades. The relative risk of death from these diseases for smokers has also remained almost unchanged over time [21]. In addition, the LE difference between smokers and non-smokers in our study was very close to that determined in a cohort study conducted in Beijing in 2000 [23]. Therefore, in the current study the changes in smoking status would have had limited influence on the estimated LE lost due to smoking and the contributions of smoking-related diseases to the life years lost. Second, although we stratified our analyses by age, sex and region, we could not make direct adjustments for socioeconomic status and unhealthy behaviors such as alcohol intake because information about these was not available. This may have caused under-estimation of the LE gap between smokers and non-smokers [24]. Third, we did not include the smoking status of ex-smoker in our study, which may have led to some under-estimation of the difference in LE. However, the influence of not considering ex-smokers separately would have been limited because smoking cessation is a relatively infrequent event in China. This is likely a consequence of the wide cultural acceptance that smoking continues to enjoy in China: most smokers quit smoking only when an illness is established [25]. Finally, we assessed only 90% of all deaths over the age of 35 years and smoking information was missing in 6% of our subjects. Although we made adjustments by reducing the base population estimate by 10% and excluding those without smoking information when calculating both death rates and smoking-specific death rates, the missing information may still have had an influence on our findings. However, the findings are unlikely to have been seriously distorted because the death patterns of deceased subjects who had no death information and of subjects who had no smoking information were probably similar to that of the subjects included in the study.

In summary, our findings suggest that respiratory disease has the greatest influence on the LE reduction associated with smoking. Thus, smoking prevention could significantly reduce deaths from respiratory disease and improve LE. This finding may have important implications in evaluating the potential effectiveness of smoking prevention programs.

## Abbreviations

LE: Life expectancy.

## Competing interests

The authors declare they have no competing interests.

## Authors’ contributions

All authors listed in our manuscript have contributed substantially to this work. BL: corresponding author, responsible for obtaining permission from all co-authors for submission of the manuscript and responsible for any changes in authorship of the manuscript; WH, JJ: joint first authors, responsible for analyzing the data, writing the manuscript, and the accuracy of the research; JL, YW, YC: responsible for study design and data collection; XZ, XZ: responsible for supervising data collection as well as quality control of the data coding; LH, HP: participated in manuscript modification before submission. All authors read and approved the final manuscript.

## Pre-publication history

The pre-publication history for this paper can be accessed here:

http://www.biomedcentral.com/1471-2458/13/1147/prepub
